# Adenosine Suppresses Cholangiocarcinoma Cell Growth and Invasion in Equilibrative Nucleoside Transporters-Dependent Pathway

**DOI:** 10.3390/ijms21030814

**Published:** 2020-01-27

**Authors:** Kornkamon Lertsuwan, Supathra Phoaubon, Nathapol Tasnawijitwong, Jomnarong Lertsuwan

**Affiliations:** 1 Department of Biochemistry, Faculty of Science, Mahidol University, Bangkok 10400, Thailand; kornkamon.ler@mahidol.edu (K.L.); aui_sp@hotmail.com (S.P.); 2 Center of Calcium and Bone Research (COCAB), Faculty of Science, Mahidol University, Bangkok 10400, Thailand; 3 Laboratory of Pharmacology, Chulabhorn Research Institute, Bangkok 10210, Thailand; nathapolt@cgi.ac.th; 4 Laboratory of Chemical Carcinogenesis, Chulabhorn Research Institute, Bangkok, 10210 Thailand

**Keywords:** purinergic signaling, cholangiocarcinoma, adenosine, equilibrative nucleoside transporters

## Abstract

Cholangiocarcinoma (CCA) is a lethal disease with increasing incidence worldwide. Previous study showed that CCA was sensitive to adenosine. Thereby, molecular mechanisms of CCA inhibition by adenosine were examined in this study. Our results showed that adenosine inhibited CCA cells via an uptake of adenosine through equilibrative nucleoside transporters (ENTs), instead of activation of adenosine receptors. The inhibition of ENTs by NBTI caused the inhibitory effect of adenosine to subside, while adenosine receptor antagonists, caffeine and CGS-15943, failed to do so. Intracellular adenosine level was increased after adenosine treatment. Also, a conversion of adenosine to AMP by adenosine kinase is required in this inhibition. On the other hand, inosine, which is a metabolic product of adenosine has very little inhibitory effect on CCA cells. This indicates that a conversion of adenosine to inosine may reduce adenosine inhibitory effect. Furthermore, there was no specific correlation between level of proinflammatory proteins and CCA responses to adenosine. A metabolic stable analog of adenosine, 2Cl-adenosine, exerted higher inhibition on CCA cell growth. The disturbance in intracellular AMP level also led to an activation of 5′ AMP-activated protein kinase (AMPK). Accordingly, we proposed a novel adenosine-mediated cancer cell growth and invasion suppression via a receptor-independent mechanism in CCA.

## 1. Introduction

Short- and long-term (trophic) purinergic signaling in human has been studied in both normal and disease conditions, including in cancers. Adenosine was shown to signal through its receptors on the cell membrane [1,2]. In addition, adenosine was reported to be transported into cytoplasm via equilibrative nucleoside transporters (ENTs) [3,4,5]. There are four subtypes of adenosine receptors in mammalian cells, namely A1, A2a, A2b and A3 [6,7]. Their activation could lead to an alteration of cAMP production [8,9,10]. Purinergic signaling regulates many physiological processes based on receptor subtypes and the concentration of their agonist(s). On the other hand, adenosine that was imported into cytoplasm via ENTs could be phosphorylated into AMP. This could disrupt AMP/ATP balance leading to an activation of AMP-activated protein kinase (AMPK) and an alteration in cell metabolism [11]. Furthermore, accumulation of intracellular adenosine could lower the ratio of S-adenosylmethionine (SAM)/S-adenosylhomocysteine (SAH) by increasing SAH level from a combination of adenosine with homocysteine. This could lead to hypomethylation of DNA [12].

A high concentration of adenosine has deleterious effects on various types of cancer, including hepatoma, pleural mesothelioma, prostate cancer and colon cancer [3,13,14,15]. Adenosine induced apoptosis and cell cycle arrest in human ovarian cancer cells (HEY, A2780 and its cisplatin-resistant subline A2780CisR) at IC_50_ around 700–900 µM. It also enhanced cisplatin sensitivity in the aforementioned cell lines [16]. Interestingly, cholangiocarcinoma (CCA) was more sensitive to adenosine than these types of cancer and had an IC_50_ around 250 µM [17].

CCA is a lethal cancer arisen from bile duct epithelial cells. The incidence of CCA is highest in the northeastern provinces of Thailand where 165.7 out of every 100,000 males were diagnosed with CCA [18]. Moreover, the increasing incidence of this cancer has been reported worldwide [19]. Therefore, novel treatment options are urgently needed due to the irresponsiveness of the disease to current chemotherapeutic drugs [20,21]. Inflammation, pesticide-induced liver and bile duct damage, hepatitis B virus infection, liver fluke infection [22,23,24,25,26] and smoking and alcohol consumption [18] also contribute to CCA progression. Several inflammatory pathways have been reported to be involved in CCA progression, such as NF-κB, Notch and iNOS pathways [27,28,29,30,31,32]. Accordingly, inflammatory pathways have also been proposed as therapeutic targets for CCA [33,34]. Among the inflammation regulating proteins, many studies have shown an involvement of protein kinase CK2 in CCA malignancy [35,36,37]. 

Recently, we have reported an inhibitory effect of adenosine on two CCA cell lines and one immortalized cholangiocyte (imCho) cell line. We have demonstrated that CCA cells were significantly more sensitive to adenosine than the imCho cells [17]. The IC_50_ values of adenosine on CCA cells were around 250 µM, much lower than on other types of cancer (1–4 mM) [3,13]. Accordingly, adenosine could be a good candidate as a novel therapeutic compound for CCA. In this study, six CCA cell lines and two imCho cell lines from both Asian and Caucasian origins were used. Among the CCA cell lines used, three CCA cell lines originated from Thai patients living in northeastern provinces where CCA is endemic.

In the present study, adenosine showed its inhibitory effects on CCA cell lines rather than imCho cells. CCA cells were shown to respond to adenosine at different degrees, leading to the classification of CCA cells into adenosine-sensitive, adenosine-resistant and unresponsive or uncalculatable (UnCal) groups. We also showed that adenosine inhibited CCA cell invasion in a receptor-independent mechanism. 

## 2. Results

### 2.1. Adenosine Suppressed CCA Cell Growth with Minimal Effect on Immortalized Cholangiocytes

**Figure 1:**
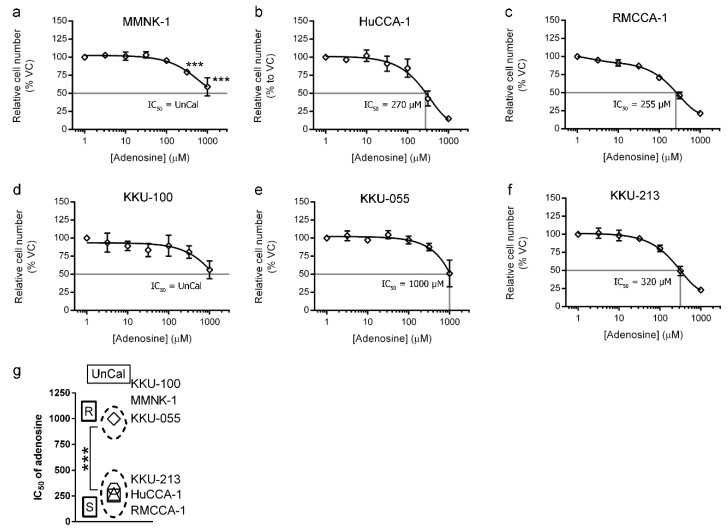
Four-day cell viability MTT assay was used to examine adenosine sensitivity on cholangiocarcinoma (CCA) and immortalized cholangiocyte (imCho) cell lines. (**a–f**) Adenosine inhibited CCA and imCho cell growth in a dose-dependent manner. (**g**) Cell lines were classified into adenosine-sensitive group, adenosine-resistant group and unresponsive or uncalculatable group, of which IC_50_ was uncalculatable. Statistical analysis in (**g**) was Student’s *t*-test. VC; vehicle control, S; sensitive, R; resistant, UnCal; uncalculatable, *** *p* < 0.001. All experiments were performed using at least three biological replicates with internal triplicate. Graphs are plotted as mean ± SD.

Cell viability assay was performed by using MTT assay to examine the sensitivity of adenosine on CCA and imCho cell growth. MMNK-1 was used to represent immortalized cholangiocyte (imCho) cell lines. HuCCA-1, RMCCA-1, KKU-100, KKU-055 and KKU-213 cell lines represented CCA cell lines originating from Thai patients. We observed that these cell lines had different sensitivities to adenosine. The imCho cell lines, MMNK-1, was more resistant as compared to most CCA cell lines, except KKU-100 (Figure 1a,d and Table 1). The IC_50_ of adenosine on MMNK-1 could not be calculated and was designated as “UnCal” since adenosine was unable to suppress the growth under 50% as compared to the vehicle control group during the 4-day period of the experiment (Figure 1a). Interestingly, other CCA cell lines, HuCCA-1, RMCCA-1 and KKU-213 were sensitive to adenosine with the IC_50_ around 250–320 µM (Figure 1b, c and f and Table 1). However, among CCA cell lines, Northeastern Thai origin cell lines, including KKU-100 and KKU-055, were resistant to adenosine. KKU-100 was also designated as “UnCal” since its IC_50_ was unable to be calculated in this experiment, and KKU-055 was resistant to adenosine with the IC_50_ at 1000 µM (Figure 1e and Table 1). According to the IC_50_, these cell lines were categorized into adenosine-sensitive, adenosine-resistant and UnCal groups according to statistical analysis (Student’s *t*-test) (Figure 1g). HuCCA-1, RMCCA-1 and KKU-213 were designated as sensitive (S). On the other hand, KKU-055 was designated as resistant (R), and KKU-100 and MMNK-1 were classified as UnCal. (Figure 1g). 

**Figure 2:**
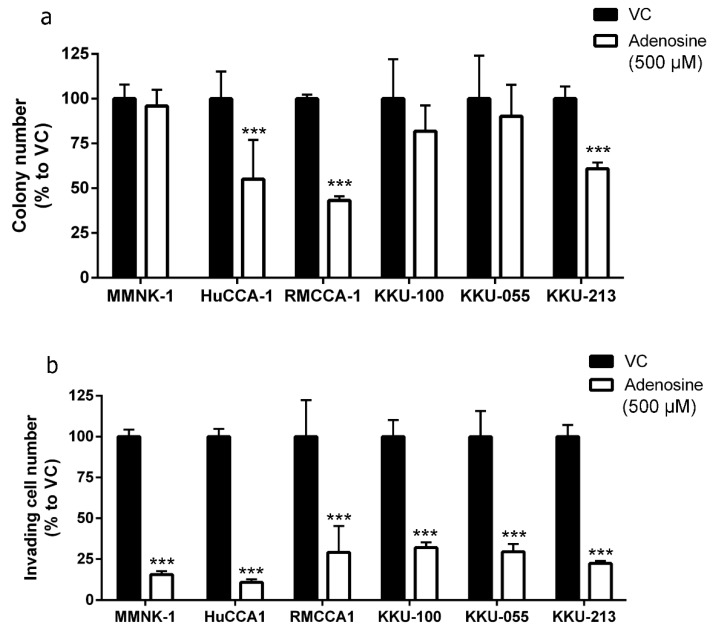
Adenosine inhibited cholangiocarcinoma (CCA) colony formation and cell invasion. (**a**) Adenosine reduced number of colony formation in adenosine-sensitive CCA cell lines after 2 weeks. (**b**) Adenosine significantly reduced number of invading cells through Matrigel in all cell lines tested, despite their sensitivity to adenosine in cell viability and colony formation assay. VC; vehicle control, *** *p* < 0.001. All experiments were performed using at least three biological replicates with internal triplicate. Graphs are plotted as mean ± SD.

In addition to MTT cell viability assay, the effect of adenosine on cell reproductive viability was also examined by utilizing clonogenic assay. The cells were treated with 500 µM adenosine for 2 weeks before the colonies were counted. Treatments were refreshed three times a week. The imCho cell line, MMNK-1, and adenosine-resistant CCA cell lines, KKU-100 and KKU-055, showed no reduction in colony number after 2 weeks of culture with 500 µM adenosine (Figure 2a). Conversely, adenosine was able to reduce the number of colonies in HuCCA-1, RMCCA-1 and KKU-213 by 50%, 60% and 45%, respectively (Figure 2a).

### 2.2. Adenosine Inhibited CCA Cell Invasion

A major problem resulting from many types of cancer, including CCA, is metastasis. We further investigated the effect of adenosine on cell invasion through Matrigel. Interestingly, adenosine reduced cell invasion in all CCA and imCho cell lines tested (Figure 2b) regardless of its sensitivity in the cell viability assay (Figure 1). In the presence of adenosine, imCho MMNK-1 cell invasion was reduced to 15.55% (Figure 2b). HuCCA-1 was the most sensitive cell line in invasion assay and was suppressed to 10.90% in the adenosine-treated group (Figure 2b). In addition, RMCCA-1, KKU-100 and KKU-055 cell invasion were suppressed to approximately 30% by adenosine. Finally, KKU-213 cell invasion was decreased to 23.36% (Figure 2b).

### 2.3. Inhibitory Effect of Adenosine on CCA Cell Growth and Invasion Was Receptor-Independent

Since adenosine could affect cells by both activating the receptors and being transported into cytoplasm via its transporters, we next investigated the mechanism underlying adenosine inhibition on CCA cells. The pan antagonists of adenosine receptors, caffeine (for A1, A2a and A2b) and CGS-15943 (for A1, A2a, A2b and A3), along with a pan inhibitor of equilibrative nucleoside transporters (ENTs), S-(4-nitrobenzyl)-6-thioinosine (NBTI), were introduced to adenosine-sensitive CCA cells with or without the presence of adenosine.

**Figure 3:**
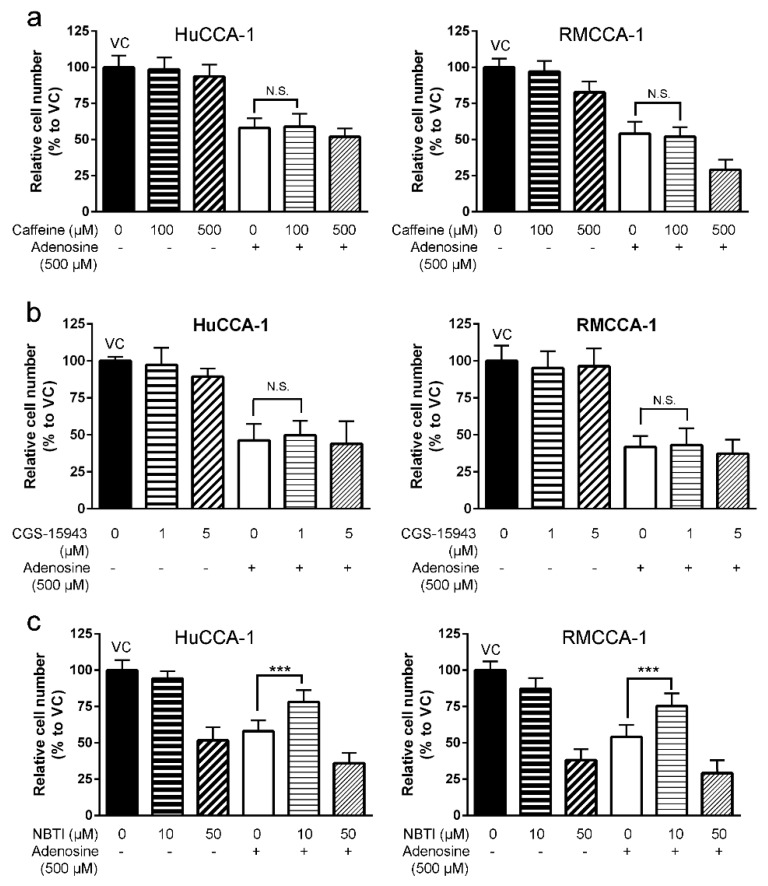
Adenosine inhibited CCA cell growth in a receptor-independent mechanism. (**a**) Caffeine, an antagonist for A1, A2a and A2b receptors, showed no significant effect on adenosine-mediated CCA cell growth suppression in viability MTT assay. (**b**) CGS-15943, a pan antagonist of adenosine receptors, showed no significant effect on adenosine-mediated CCA cell growth suppression in viability MTT assay. (**c**) Inhibitory effect of adenosine on cell growth subsided when 10 µM (4-nitrobenzyl)-6-thioinosine (NBTI), a broad inhibitor of equilibrative nucleoside transporters (ENTs), was applied 1 h prior to adenosine treatment. VC; vehicle control, N.S.; not significant, *** *p* < 0.001. All experiments were performed using at least three biological replicates with internal triplicate. Graphs are plotted as mean ± SD.

We demonstrated that 500 µM adenosine inhibited cell growth to 55% and 50% in HuCCA-1 and RMCCA-1, respectively (Figure 3a). Interestingly, addition of caffeine (Figure 3a) or CGS-15943 (Figure 3b) to adenosine was unable to reduce an inhibitory effect of adenosine on cell viability (MTT assay) in these three cell lines. In contrast, introduction of 10 µM NBTI was able to reduce inhibitory effect of adenosine on all cell lines tested (Figure 3c). Cell viability was increased in CCA cells treated with adenosine together with NBTI as compared to CCA cells treated with adenosine alone from approximately 50% to 75% in both HuCCA-1 and RMCCA-1 (Figure 3c).

**Figure 4:**
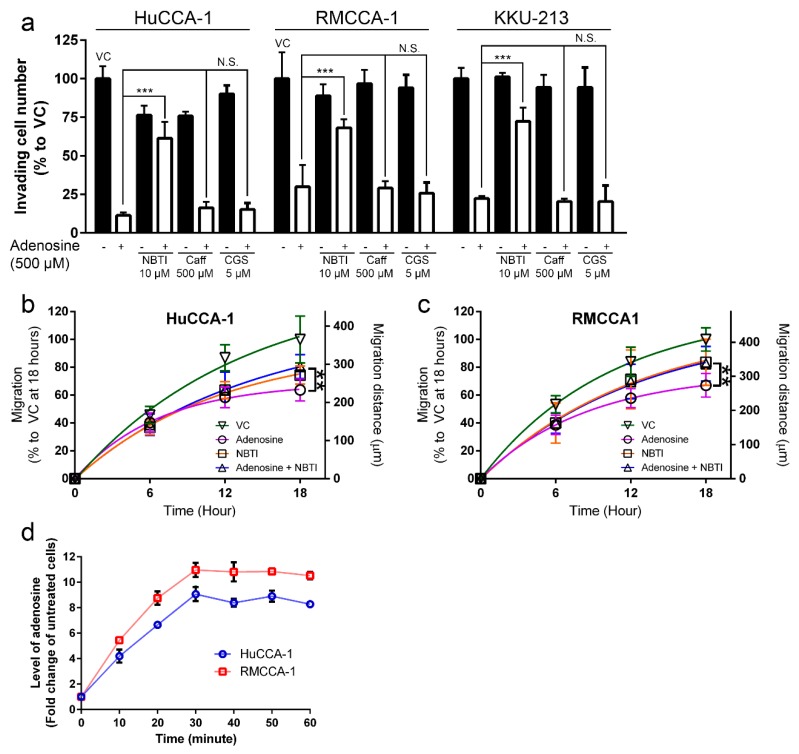
Adenosine inhibited cholangiocarcinoma (CCA) cell motility in a receptor-independent mechanism. (**a**) Adenosine suppressed CCA cell invasion through Matrigel in a receptor-independent mechanism. Caffeine and CGS-15943, pan antagonists of adenosine receptors, showed no significant effect on adenosine-mediated CCA cell invasion suppression. Inhibitory effect of adenosine on cell invasion was reduced when 10 µM (4-nitrobenzyl)-6-thioinosine (NBTI), a broad inhibitor of equilibrative nucleoside transporters (ENTs), was applied 1 h prior to adenosine treatment. (**b**–**c**) Adenosine also suppressed CCA cell migration in 2D culture in a receptor-independent mechanism. NBTI at 10 µM was able to reduce adenosine-mediated inhibitory effect on CCA cell migration in wound-healing assay. (**d**) Intracellular adenosine level increased after 500 µM adenosine treatment. VC; vehicle control, Caff; caffeine, CGS; CGS-15943, N.S.; not significant, ** *p* < 0.01, *** *p* < 0.001. All experiments were performed using at least three biological replicates with internal triplicate. Graphs are plotted as mean ± SD.

Furthermore, both 500 µM caffeine and 5 µM CGS-15943 could not reduce an inhibitory effect of adenosine on CCA cell invasion in all CCA cell lines tested (Figure 4a). The invading cell number in caffeine/CGS-15943 plus adenosine-treated group remained the same as in the vehicle control plus adenosine-treated group in all cell lines tested (Figure 4a). Conversely, 10 µM NBTI was able to significantly alleviate an inhibitory effect of adenosine on CCA cell invasion in all cell lines tested. Inhibitory effects of adenosine on CCA cell invasion was recovered from 11.4% to 61.4% in HuCCA-1, from 30.0% to 68.2% in RMCCA-1 and from 22.4% to 72.3% in KKU-213 in the presence of NBTI (Figure 4a). In addition, the results showed that NBTI also suppressed adenosine effects on cell migration in 2D tissue culture plate. The inhibitory effect was lowered by approximately 20% after 18 h of adenosine treatment together with NBTI (Figure 4b,c). 

In addition, we demonstrated that intracellular adenosine level was increased after 500 µM adenosine treatment in all CCA cell lines tested. Intracellular adenosine was increased to 9.1-, 9.5- and 11.0-fold higher than untreated cells before reaching a plateau at 30 min (Figure 4d). Therefore, we concluded that adenosine suppressed CCA cell invasion and migration via a receptor-independent but transporter-dependent mechanism.

### 2.4. Correlation between Level of Inflammatory Proteins and Adenosine Sensitivity in CCA Cell Lines was not Observed

**Figure 5:**
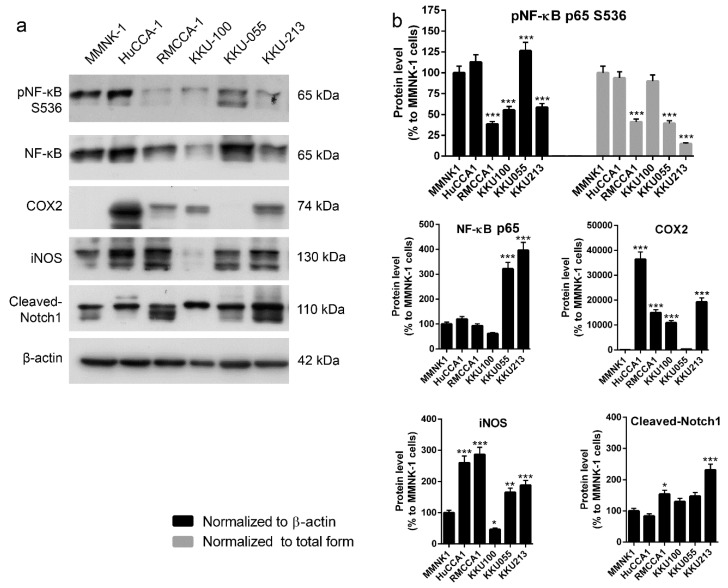
Levels of proteins in inflammatory pathways in cholangiocarcinoma (CCA) and immortalized cholangiocyte cell lines were investigated. (**a**) There is no correlation between level of inflammatory proteins and adenosine sensitivity in CCA cell lines. (**b**) Semiquantitative data of protein levels in (**a**) * *p* < 0.05, ** *p* < 0.01, *** *p* < 0.001. All experiments were performed using at least three biological replicates with internal triplicate. Graphs are plotted as mean ± SD.

Our data demonstrated that imCho cell lines (MMNK-1) and CCA cell lines (KKU-100 and KKU-055) were more resistant to adenosine as compared to other CCA cell lines (Figure 1 and Figure 2). We further investigated the underlying mechanism that makes most CCA cell lines sensitive to adenosine; a few CCA cell lines and imCho cell lines were resistant. The levels of proinflammatory molecules were investigated because inflammation was shown to be involved in CCA progression and aggressiveness [38,39,40]. Levels of nuclear factor-κB (NF-κB), cyclooxygenase 2 (COX2), inducible nitric oxide synthase (iNOS) and Notch1 were examined in five CCA cell lines and one imCho cell lines (Figure 5a,b). The level of all aforementioned proteins showed no specific correlation to adenosine sensitivity in CCA cell lines. Furthermore, CCA cell lines that were adenosine-resistant grew slower and had longer doubling time as compared to CCA cell lines that were sensitive to adenosine (Table 1). Doubling times for KKU-100 and KKU-055, which were adenosine-resistant, were approximately 70 and 97 h, respectively; other CCA cell lines had shorter doubling times of approximately 40 h (Table 1). Nonetheless, the imCho cell line, MMNK-1, was resistant to adenosine but had doubling times of around 40 h (Table 1), which is comparable to adenosine-sensitive CCA cell lines. These data suggested that adenosine did not solely inhibit any fast-growing cells, but its inhibitory effects depended on a specific molecular mechanism not yet revealed.

### 2.5. A Conversion of Adenosine to AMP Is Required for the Inhibition

We have demonstrated that adenosine treatment led to increased intracellular adenosine and could potentially be transported into CCA cells by ENTs (Figure 3 and Figure 4). Therefore, we further investigated the possible metabolic phases of adenosine once it was transported into CCA cells. In normal cells, intracellular adenosine could be converted to AMP by the activity of adenosine kinase to regenerate the energy carrier molecules (ADP and ATP, subsequently) and to serve as a precursor for nucleotide synthesis [41]. On the other hand, adenosine could also be deaminated by adenosine deaminase to inosine, which can also be used as another precursor in nucleotide synthesis [41]. Hence, to validate the roles of adenosine kinase, metabolic stable adenosine (2Cl-adenosine) and inosine were further tested to elucidate the potential metabolic phases of intrasellar adenosine in adenosine-treated CCA cells. 

**Figure 6:**
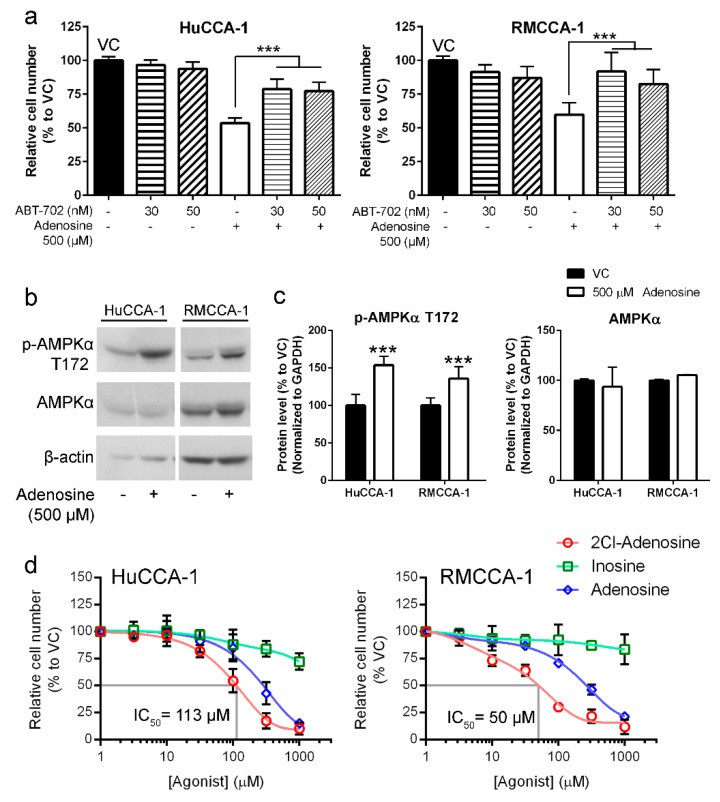
Adenosine inhibited CCA cells through a conversion to AMP. (**a**) MTT assay showed CCA cell viability after 24 h adenosine treatment with or without ABT-702, an inhibitor of AMP-producing enzyme: adenosine kinase. ABT-702 was able to reduce adenosine’s inhibitory effect. (**b**) Adenosine treatment increased phosphorylated form of AMPKα after 24 h of the treatment. (**c**) Semiquantitative data of protein levels in (**b**). (**d**) 2-Chloroadenosine (2Cl-adenosine) also inhibited CCA cell lines in MTT viability assay but inosine had only little effect on CCA cell viability. VC; vehicle control, *** *p* < 0.001. All experiments were performed at least three biological replicates with internal triplicate. Graphs were plotted as mean ± SD.

Adenosine kinase inhibitor ABT-702 was used in the experiment to determine the requirement of this enzyme in adenosine-mediated CCA cell suppression. Our results showed that ABT-702 reduced an inhibitory effect of adenosine on CCA cell viability in MTT assay (Figure 6a). Higher cell viability was observed in HuCCA-1 (from 53.6% in adenosine-treated group to 78.7% with a presence of 30 µM ABT-702) and RMCCA-1 (from 59.9% in adenosine-treated group to 91.8% with a presence of 30 µM ABT-702) (Figure 6a). Therefore, the activity of adenosine kinase is required for adenosine-induced CCA cell suppression. As many studies showed that phosphorylated AMPKα was increased when the ratio between intracellular AMP + ADP and ATP (AMP + ADP/ATP) was higher [42], the increased level of AMP as indicated by the increased level of phosphorylated AMPKα was examined. Our results showed that phosphorylated AMPKα was increased to 153% and 135% in HuCCA-1 and RMCCA-1, respectively (Figure 6b,c). These results indicated that adenosine-induced CCA cell suppression mechanism requires adenosine kinase activity. In other words, adenosine, which was imported into CCA cells, was phosphorylated into AMP as indicated by the requirement of adenosine kinase activity and the increase of phosphorylated AMPKα. 

Furthermore, to confirm the AMP-mediated mechanism in adenosine-induced CCA cell inhibition, 2-chloroadenosine (2Cl-adenosine) and inosine were tested for their potential inhibitory effect on CCA cells. 2Cl-adenosine is a metabolically stable analog of adenosine, which has been shown to preferably change to AMP rather than inosine. Our results revealed that 2Cl-adenosine had higher inhibitory effect on CCA cells as compared to adenosine, suggesting the preferable path of adenosine-induced CCA cell inhibition via AMP formation (Figure 6d). Its IC_50_ values on HuCCA-1 and RMCCA-1 were 113 and 50 µM, respectively (Figure 6d). In contrast, inosine had only minimal inhibitory effect on CCA cells. Inosine concentration as high as 1000 µM reduced HuCCA-1 and RMCCA-1 only to 72.2% and 83.6% (Figure 6d). The results indicated that inosine was not likely to play a role in CCA cell suppression by adenosine. 

## 3. Discussion

Five CCA and one imCho cell lines were examined for their sensitivity to adenosine and was categorized into three groups: adenosine-sensitive group (IC_50_ between 250–320 µM), adenosine-resistant group (IC_50_ 1000 µM) and uncalculatable (UnCal) group. The UnCal groups were the cells for which an IC_50_ was unable to be calculated because adenosine was unable to suppress the growth under 50% as compared to the vehicle control group during the 4-day period of the experiment. Interestingly, adenosine was also able to suppress cell invasion through Matrigel in all cell lines regardless to their sensitivity in cell viability MTT assay. We further investigated the potential molecular pathways underlying this inhibition and have demonstrated that adenosine inhibited CCA cells in a receptor-independent mechanism. Some of these cell lines including HuCCA-1, RMCCA-1 and MMNK-1 were reported to not express adenosine receptor genes, [17] while other cell lines expressed some adenosine receptor genes (Figure S1 and Table S1). Furthermore, expression of ENPP1-3 and ENTPD1-3 (CD39 family), which convert ATP to AMP [43,44], was ubiquitous. At least one of the ENPP or ENTPD members was expressed on CCA and imCho cell lines (Figure S1 and Table S1). Accordingly, the presence of adenosine receptors and adenosine-producing enzymes and adenosine sensitivity did not correlate. In addition, pan adenosine receptor antagonist, caffeine and CGS-15943 failed to rescue CCA cells from adenosine inhibitory effects (Figure 3 and Figure 4). Previous study showed that adenosine could also be transported into cytoplasm via equilibrative nucleoside transporters (ENTs). By using ENTs inhibitor, S-(4-nitrobenzyl)-6-thioinosine (NBTI), our results showed that NBTI was able to rescue CCA from adenosine (Figure 3 and Figure 4). This suggested the transporter-dependent cancer inhibition, similar to what has previously been reported in colon and cervical cancers [3,4]. Since the activity of ENTs was required in CCA cell inhibition by adenosine, intracellular level of adenosine in CCA cells after adenosine treatment was examined. The results showed that exogenous treatment of adenosine of CCA cells increased the level of intracellular adenosine (Figure 4e).

It has been reported that intracellular adenosine could be converted to other metabolites, including AMP and inosine. Adenosine could be phosphorylated to AMP by the activity of adenosine kinase, or it could be converted to inosine by the activity of adenosine deaminase as mentioned previously [41]. Accordingly, the potential metabolic change of adenosine in adenosine-mediated CCA cell suppression was elucidated. As the major enzyme to phosphorylate adenosine to AMP, the contribution of adenosine kinase in adenosine-mediated CCA cell suppression was examined. The results showed that adenosine’s inhibitory effects on CCA cells were subsided in the presence of adenosine kinase inhibitor, indicating its important role in adenosine-mediated CCA cell suppression (Figure 6a). Further, the increased level of intracellular AMP was also indirectly shown by the level of phosphorylated AMPKα in CCA cells exposed to adenosine. These suggested the potential mechanism of CCA cell suppression by adenosine through AMP production by adenosine kinase. To further confirm this speculation, 2Cl-adenosine, which is a metabolically stable analog of adenosine, was used as representative of adenosine that was mainly converted to AMP as previously reported [45]. Our results showed a higher inhibitory effect of 2Cl-adenosine on CCA cell viability as compared to adenosine. In addition, inosine, another metabolic product of adenosine, had minimal effect on CCA cell viability. These data suggested that adenosine exerted its inhibitory effect when it is not metabolically converted into inosine, but rather converted to AMP. 

Generally, chemotherapeutic drugs tend to be more effective on fast-growing cells regardless of cell type (cancerous or noncancerous). This causes deleterious side effects, including hair loss, nausea and diarrhea. Interestingly, adenosine had much less deleterious effects on noncancerous cell growth despite their fast-growing characteristic (Figure 1a and Table 1). Therefore, adenosine was shown to be a strong candidate for the novel therapeutics for CCA. Further experiments in animals are needed to assess the tolerance and side effects of adenosine in vivo. 

In summary, our study revealed the therapeutic potential and differential responses of adenosine on CCA cells. The novel adenosine-mediated cancer cell suppression through a receptor-independent but nucleoside-transporter-dependent mechanism in CCA cells was elaborated. Extracellular adenosine treatment led to increased intracellular adenosine, which was later phosphorylated to AMP by adenosine kinase and an activation of AMPK.

## 4. Materials and Methods 

### 4.1. Cell Culture

Characteristics of all cell lines used in this study are described in Table 1. Three CCA cell lines, KKU-100, KKU-055 and KKU-213, were established as previously described [46] and were purchased from Japanese Collection of Research Bioresources (JCRB) Cell Bank (Osaka, Japan). The imCho cell line, MMNK-1, was also purchased from JCRB Cell Bank. HuCCA-1 was established and kindly provided by Stitaya Sirisinha at Chulabhorn Research Institute [47]. RMCCA-1 cell line was established from a peripheral CCA specimen surgically obtained from a Thai patient and was kindly provided by Rutaiwan Tohtong at Mahidol University [48]. All cell lines were maintained in Dulbecco’s modified Eagle’s medium (DMEM) (SH30243.02, Hyclone, Pittsburgh, PA, USA) supplemented with 10% fetal bovine serum (FBS) (10270-106 Brazil origin, Gibco, Grand Island, NY, USA) and 1% MEM non-essential amino acid (1140-050, Gibco). All media were supplemented with 1% penicillin/streptomycin (15140-122, Gibco). All cell lines were maintained at 37 °C with 5% CO_2_.

### 4.2. Cell Viability MTT Assay

Cells were plated in tissue culture treated 96-well plates. After overnight adhesion to the plate, the treatment groups were incubated with adenosine (A4036, Sigma Aldrich) at 3.16 to 1000 µM with a half log increment. Media were changed and new treatments were added on day 2. After 4 days, MTT reagent (M6494, Fisher Scientific, Hampton, NH, United States) was added to the final concentration of 0.5 mg/mL in 100 µL culture media and incubated for 2.5 h at 37 °C. Fifty microliters of stop solution (10% SDS in 50% dimethylformamide in dH_2_O) was added and mixed thoroughly before reading the absorbance at 560 nm on Multimode Plate Reader Victor Nivo (Perkin Elmer). The following reagents, S-(4-nitrobenzyl)-6-thioinosine (NBTI) (sc-200117, Santa Cruz Biotechnology, Dalla, TX, USA), caffeine (C 5-3, Sigma-Aldrich, St. Louis, MO, USA), CGS-15943 (C199, Sigma-Aldrich) or ABT-702 (2372, Tocris, Bristol, UK), were added according to the experimental design.

### 4.3. Clonogenic Assay

Cells were plated at 500 cells per well in tissue-culture-treated 24-well plate and allowed to adhere overnight. Adenosine (A4036, Sigma Aldrich) at 500 µM was added and incubated for 2 weeks. Media and treatment were changed regularly, three times per week. After 2 weeks, cells were stained with 0.5% (*w*/*v*) crystal violet in 12% (*v*/*v*) glutaraldehyde in water for 30 min at 23 °C. Cells were rinsed with dH_2_O and manually counted under microscope. A group of 100 cells or more was counted as one colony.

### 4.4. Cell Invasion Assay

Eight-micrometer polycarbonate membrane transwell inserts (353097, Falcon, New York, NY, USA) were coated with 50 µL of a 1:10 mixture of Matrigel™ (356234, BD Biosciences, San Jose, CA, USA) in serum-free medium. Then, it was allowed to congeal at 37 °C for 30 min. A total of 5 × 10^4^ cells were added to the insert in serum-free medium. The bottom well was filled with complete medium. Treatment of 500 µM adenosine (A4036, Sigma Aldrich) was added in both top and bottom chambers. Ten micromolars of S-(4-nitrobenzyl)-6-thioinosine (NBTI) or 500 µM of caffeine or 5 µM of CGS-15943 were added 1 h prior to adenosine (A4036, Sigma Aldrich) if they were required for the experiment. Cells were incubated for 24 h in a 37 °C, humidified incubator with 5% CO_2_. The chambers were swabbed to remove cells and Matrigel™ remaining on the top of the membrane. Cells on the lower side of the membrane were fixed in methanol for 5 min and stained with 0.5% (*w*/*v*) crystal violet in 12% glutaraldehyde in water for 15 min. Following a brief dH_2_O wash, cells were counted using Nikon Eclipse T2S phase contrast inverted fluorescence microscope.

### 4.5. Intracellular Adenosine Assay

Cells were plated at 2 × 10^4^ cells per well in tissue culture treated 24-well plate and allowed to adhere overnight. Adenosine at 500 µM was added and incubated for 10, 20, 30, 40, 50 and 60 min before trypsinization. Cell pellets were sonicated to break cell membranes and then centrifuged at 12,000× *g* for 10 min at 4 °C. Supernatant was collected and adenosine levels were measured by using Adenosine Assay Kit (Fluorometric) (ab211094, Abcam, Cambridge, MA, USA) according to manufacturer’s protocol.

### 4.6. Western Blot Analysis

A total of 25 µg of proteins was separated electrophoretically and transferred to nitrocellulose membrane (10600003, GE, Boston, MA, USA) at 23 °C. Membranes were blocked in blocking buffer (4% BSA *w*/*v* in TBST), and then incubated with primary antibodies overnight at 4 °C on a rocking shaker. All antibodies were diluted in blocking buffer (4% BSA in 0.1% TBST). Primary antibodies included, 1:1500 phospho-NF-κB p65 (Ser536) (13346, Cell Signaling Technology, Danvers, MA, USA), 1:1500 NF-κB p65 (4764, Cell Signaling Technology), 1:1500 COX2 (12282, Cell Signaling Technology), 1:1500 iNOS (13120, Cell Signaling Technology), 1:1500 cleaved Notch1 (4147, Cell Signaling Technology), AMPKα (5832, Cell Signaling Technology), phospho-AMPKα T172 (2535, Cell Signaling Technology), 1:5000 GAPDH (8884, Cell Signaling Technology) and 1:5000 β-actin (A2066, Sigma Aldrich). Membranes were incubated for 75 min on a rocking shaker at 23 °C with secondary antibodies. Secondary antibodies included 1:5000 goat anti-rabbit IgG conjugated with horseradish peroxidase (7074, Cell Signaling Technology) and 1:5000 horse anti-mouse IgG conjugated with horseradish peroxidase (7076, Cell Signaling Technology). Signal was visualized by autoradiography using enhanced chemiluminescence (ECL) and exposed to Hyperfilm (28906838, GE Healthcare, Boston, MA, USA). Band intensity was analyzed by using ImageJ software (version 1.52a, National Institute of Health, Bethesda, MD, USA).

### 4.7. Reverse Transcriptase Polymerase Chain Reaction (RT-PCR)

Total RNA was extracted using TRIzol reagent (15596-026, Life Technologies, Carlsbad, CA, USA). Following the extraction, RNA was treated with RNAse-free DNase I (04716728001, Roche, Basel, Switzerland). RNA quality and quantity were analyzed spectrophotometrically and electrophoretically. RNA was stained with NovelJuice (LD001-1000, Gendirex, Taoyuan, Taiwan). Following quality and quantity verification, 5 μg of total RNA was reverse transcribed using Maloney murine leukemia virus (M-MLV) reverse transcriptase (28025-013, Life Technologies). Polymerase chain reaction (PCR) was carried out using helixamp Taq (TBF500N, Nanohelix, Daejeon, South Korea) for 32 cycles with 2 mM MgCl_2_, 0.2 mM dNTP, 1 μM of forward and reverse primers, 0.025 U/μL of Taq polymerase. Primer sequences are shown in Table S2.

### 4.8. Statistical Analysis

Data were graphed as mean ± standard deviation (SD). Statistical analyses were performed using ANOVA with Dunnett’s test unless otherwise stated. All experiments were performed in at least biological triplicate.

## 5. Conclusions

Adenosine inhibited CCA cell growth and motility in a receptor-independent but ENT-dependent mechanism. The treatment led to an increased intracellular adenosine level, which was further changed to AMP by the activity of adenosine kinase. This conversion of adenosine to AMP is required for the inhibition. This led to an activation of AMPK. Our study provided a novel mechanism underlying CCA cell suppression by adenosine, indicating the therapeutic potential of adenosine for CCA. 

## Figures and Tables

**Table 1:** IC_50_ and pIC_50_ of the adenosine on cholangiocarcinoma (CCA) and immortalized cholangiocyte (imCho) cell lines. UnCal; uncalculatable.

Cell Line	Cell Type	Type	Origin	Doubling Time (h)	Sex	IC_50_ of the Adenosine (µM)	pIC_50_
MMNK-1	imCho		Japanese	41.43	Male	UnCal	UnCal
HuCCA-1	CCA	intrahepatic	Thai	43.95	Male	270	3.57
RMCCA-1		peripheral		49.40	Male	255	3.59
KKU-100		extrahepatic	Thai (Northeastern)	70.48	Female	UnCal	UnCal
KKU-055		intrahepatic		97.40	Male	1000	3.00
KKU-213		intrahepatic		34.40	Male	320	3.49

## Supplementary Materials

Supplementary materials can be found at https://www.mdpi.com/1422-0067/21/3/814/s1.

## Abbreviations

CCA	Cholangiocarcinoma
ENTs	Equilibrative nucleoside transporters
imCho	Immortalized cholangiocytes
NBTI	S-(4-Nitrobenzyl)-6-thioinosine
