# Supplementary material for: Adenosine Suppresses Cholangiocarcinoma Cell Growth and Invasion in Equilibrative Nucleoside Transporters-Dependent Pathway
Source: Int J Mol Sci. 2020 Jan 27;21(3):814. doi: 10.3390/ijms21030814 (PMC7037771; doi:10.3390/ijms21030814)
Supplement: Supplementary file 1 [file ijms-21-00814-s001.zip › KL_Supplementary Table S1.docx]

**Table S1** Transcriptional profile of adenosine receptors on CCA cells. Transcriptional profile obtained from reverse transcription PCR. + indicates the presence of the specified gene expression. Expression data of HuCCA-1, RMCCA-1 and MMNK-1 were previously published [17]

| **Cell line** | **ADORA Subtypes** | | | | **ENPP** | | | **ENTPD** | | |
| --- | --- | --- | --- | --- | --- | --- | --- | --- | --- | --- |
|  | 1 | 2a | 2b | 3 | 1 | 2 | 3 | 1 | 2 | 3 |
| MMNK-1 | - | - | - | - | - | + | - | - | - | - |
| HuCCA-1 | - | - | - | - | - | + | - | - | - | - |
| RMCCA-1 | - | - | - | - | - | + | - | - | - | - |
| KKU-100 | - | - | + | - | - | + | - | + | - | - |
| KKU-055 | - | + | + | - | + | - | - | - | + | - |
| KKU-213 | - | - | + | - | + | - | - | - | + | + |

17. Lertsuwan, J.; Ruchirawat, M. Inhibitory effects of ATP and adenosine on cholangiocarcinoma cell proliferation and motility. *Anticancer Res* **2017**, *37*, 3553-3561, doi:10.21873/anticanres.11725.
