# Supplementary material for: Adenosine Suppresses Cholangiocarcinoma Cell Growth and Invasion in Equilibrative Nucleoside Transporters-Dependent Pathway
Source: Int J Mol Sci. 2020 Jan 27;21(3):814. doi: 10.3390/ijms21030814 (PMC7037771; doi:10.3390/ijms21030814)
Supplement: Supplementary file 1 [file ijms-21-00814-s001.zip › KL_Supplementary Figure S1.docx]

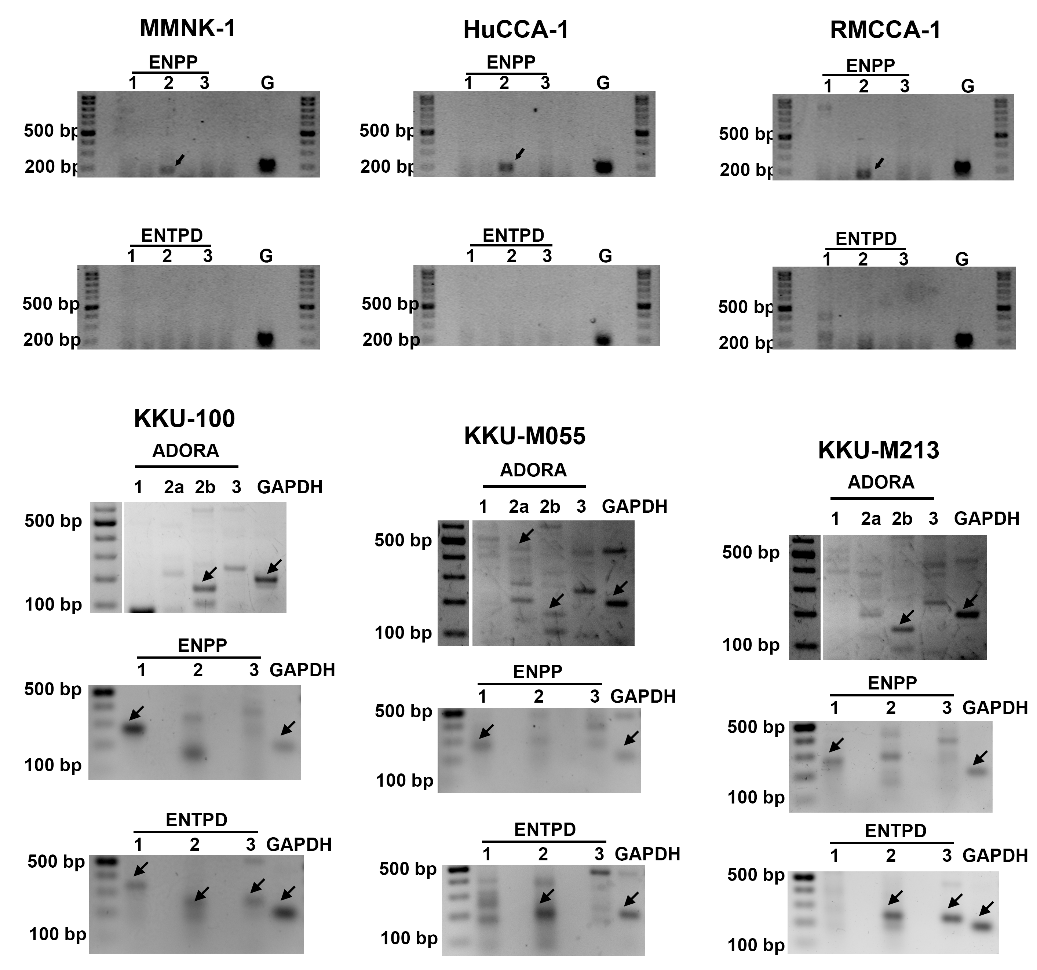


**Figure S1** Basal level of expression of genes encoding for adenosine receptors and ectonucleotidase enzymes. Reverse transcription PCR showed a presence of ADORA, ENTPD and ENPP genes, which encode adenosine receptors and ectonucleotidase enzyme, in cholangiocarcinoma cell lines and immortalized cholangiocyte cell lines. Arrow indicates the correct product size. Gel images are the represents of 3 biological replicates
