# Supplementary material for: Adenosine Suppresses Cholangiocarcinoma Cell Growth and Invasion in Equilibrative Nucleoside Transporters-Dependent Pathway
Source: Int J Mol Sci. 2020 Jan 27;21(3):814. doi: 10.3390/ijms21030814 (PMC7037771; doi:10.3390/ijms21030814)
Supplement: Supplementary file 1 [file ijms-21-00814-s001.zip › KL_Supplementary Table S2.docx]

**Table S2** Primer sequences for purinergic receptors genes. Rev: Reverse, For: Forward

| Gene | | Primer sequences | Expected product size  (base pair) |
| --- | --- | --- | --- |
| A1 For | CAACATTGGGCCACAGACCT | | 222 |
| A1 Rev | ATAGGGGTCAGTCCCACCAC | |  |
| A2A For | ATCGCCATTGACCGCTACAT | | 493 |
| A2A Rev | AGTCGGGGCAGAAGAAAGTG | |  |
| A2b For | GGGCTTCTGCACTGACTTCT | | 175 |
| A2b Rev | AGCAATGACCCCTCTTGCTC | |  |
| A3 For | CACCTGTGATGAGCCCTTTCT | | 577 |
| A3 Rev | GTGAGTGGTGACCCTCTTGT | |  |
| ENPP1 For | CCTTGCAGAGGGTTGATGGT | | 275 |
| ENPP1 Rev | TGGTTCCCGGCAAGAAAGAT | |  |
| ENPP2 For | CAAGGGCAGGTGCTTTGAAC | | 256 |
| ENPP2 Rev | CCAATGCGACTCTCCTTTGC | |  |
| ENPP3 For | GCCAAAGCGACTGCACTATG | | 159 |
| ENPP3 Rev | AGAAAGATAGCCTCCATGCTCC | |  |
| ENTPD1 For | GAGGAGCCTCAGCAACTACC | | 331 |
| ENTPD1 Rev | CCAGAGTGCCTGATCCTTCC | |  |
| ENTPD2 For | AGGAGAACGACACAGGCATT | | 218 |
| ENTPD2 Rev | CTCTGGATTGGTCAGGTTGAGC | |  |
| ENTPD3 For | CAAGGCCCTCTACCGAACTC | | 257 |
| ENTPD3 Rev | TGGAGATTCCAGAGCCTTTCAC | |  |
| GAPDH For | CACCGTCAAGGCTGAGAACG | | 223 |
| GAPDH Rev | GACGAACATGGGGGCATCAG | |  |
